# *Culex erythrothorax* (Diptera: Culicidae): Activity periods, insecticide susceptibility and control in California (USA)

**DOI:** 10.1371/journal.pone.0228835

**Published:** 2020-07-10

**Authors:** Allen T. Esterly, Dereje Alemayehu, Benjamin Rusmisel, John Busam, Theresa L. Shelton, Tina Sebay, Nayer Zahiri, Joseph W. Huston, Ryan J. Clausnitzer, Eric J. Haas-Stapleton

**Affiliations:** 1 Alameda County Mosquito Abatement District, Hayward, California, United States of America; 2 San Mateo County Mosquito and Vector Control District, Burlingame, California, United States of America; Al-Azhar University, EGYPT

## Abstract

The mosquito *Culex erythrothorax* Dyar is a West Nile virus (WNV) vector that breeds in wetlands with emergent vegetation. Urbanization and recreational activities near wetlands place humans, birds and mosquitoes in close proximity, increasing the risk of WNV transmission. Adult *Cx*. *erythrothorax* abundance peaked in a wetland bordering the San Francisco Bay of California (USA) during the first 3 hours after sunset (5527 ± 4070 mosquitoes / trap night) while peak adult *Culex tarsalis* Coquillett abundance occurred during the subsequent 3 h period (83 ± 30 *Cx*. *tarsalis*). When insecticide resistance was assessed using bottle bioassay, *Cx*. *erythrothorax* was highly sensitive to permethrin, naled, and etofenprox insecticides compared to a strain *of Culex pipiens* that is susceptible to insecticides (LC_50_ = 0.35, 0.71, and 4.1 μg/bottle, respectively). The *Cx*. *erythrothorax* were 2.8-fold more resistant to resmethrin, however, the LC_50_ value was low (0.68 μg/bottle). Piperonyl butoxide increased the toxicity of permethrin (0.5 μg/bottle) and reduced knock down time, but a higher permethrin concentration (2.0 μg/bottle) did not have similar effects. Bulk mixed-function oxidase, alpha-esterase, or beta-esterase activities in mosquito homogenates were higher in *Cx*. *erythrothorax* relative to the *Cx*. *pipiens* susceptible strain. There was no difference in the activity of glutathione S-transferase between the two mosquito species and insensitive acetylcholine esterase was not detected. Larvicides that were applied to the site had limited impact on reducing mosquito abundance. Subsequent removal of emergent vegetation in concert with larvicide applications and reduced daily environmental temperature substantially reduced mosquito abundance. To control *Cx*. *erythrothorax* in wetlands, land managers should consider vegetation removal so that larvicide can efficiently enter the water. Vector control agencies may more successfully control adult viremic *Cx*. *erythrothorax* that enter nearby neighborhoods by applying adulticides during the 3 h that follow sunset.

## Introduction

*Culex erythrothorax* Dyar (Diptera: Culicidae), commonly known as the tule mosquito, is endemic to the western southwestern states of the United States [1]. The larvae breed in heavily vegetated regions of shallow ponds and can be highly abundant in marsh habitats that contain dense clusters of *Schoenoplectus spp* (common tule), *Typha spp*. (bulrush), or *Myriophyllum aquaticum* (parrot feather) [2–4]. Unlike many species of mosquitoes, adult *Cx*. *erythrothorax* do not disperse distantly from where they emerge [3, 5, 6]. The time of host-seeking for *Culex tarsalis* Coquillett, another mosquito species found in marsh habitats, occurs 1–4 h after sunset [7, 8]. The time of day that *Cx*. *erythrothorax* is most likely to be actively flying and would be best controlled by insecticides is not reported. Larvicide applications to constructed marsh habitats can markedly reduce the abundance of adult *Cx*. *erythrothorax* [9]. However, the reduction may be minimal if dense aquatic vegetation limits the penetration of larvicide into the water column and adult mosquitoes immigrate from nearby sites [5]. We describe herein the impact of larvicide applications and removing emergent vegetation on *Cx*. *erythrothorax* abundance.

Adult female *Cx*. *erythrothorax* aggressively bite mammals and birds, and may transmit West Nile virus (WNV) [6, 10, 11]. While about 80% of WNV infections in humans are apparently asymptomatic, serious neuroinvasive disease develops in less than 1% of infected persons [12]. The greatest risk for human exposure to WNV is thought to come from biting *Culex pipiens* Linnaeus and *Culex quinquefasciatus* Say. However, approximately 10-fold more *Cx*. *erythrothorax* that were collected in a marsh habitat abutting a suburban landscape contained human blood compared to *Cx*. *quinquefasciatus* that were collected in the same traps [5]. Thus, the risk of human exposure to WNV by *Cx*. *erythrothorax* may increase as people seek to reside near and recreate in marsh habitats. This mosquito may also maintain the transmission of WNV among birds in marsh habitats, with the cooccurring *Cx*. *tarsalis* transmitting the virus to humans. When larvicides are ineffective in controlling *Cx*. *erythrothorax* larvae, insecticides that target adult mosquitoes may be employed to interrupt arbovirus transmission cycles.

Pyrethroid and organophosphate insecticides are used by public health agencies to control adult mosquitoes [13]. The present study describes the susceptibility of adult female *Cx*. *erythrothorax* to permethrin, resmethrin, etofenprox and naled insecticides. Mosquitoes can increase the quantity or activity of enzymes that metabolize insecticides, rendering them inactive and unable to kill the exposed insects. Herein, we examined the activity of alpha- and beta-esterase, glutathione S-transferase (GST), mixed-function oxidase (MFO), and insensitive acetylcholine esterase enzymes in mosquitoes. Esterases are a large family of enzymes that hydrolyze ester bonds within insecticides [14]. Insecticides are oxidized by MFO while GST conjugate glutathione to insecticides, thereby increasing their solubility in water and rate of excretion from the insect [15–17]. Piperonyl butoxide (PBO) is a synergist that can be included with insecticides to inhibit MFO and increase the efficacy of insecticides [18].

## Materials and methods

### Mosquito and metrological data collection

Adult *Culex erythrothorax* mosquitoes were collected over night from 2016–2019 at the Hayward Marsh, a 0.13 km^2^ freshwater marsh that abuts the San Francisco Bay, CA USA (GPS coordinates: 37.629986, -122.141174) using Encephalitis Vector Survey traps (EVS; BioQuip, Rancho Dominguez, CA) or a Collection Bottle Rotator Trap (CBRT; John W. Hock Company, Gainesville, FL) that were baited with dry ice. A scientific collection permit was not required because the collections were made by a mosquito abatement district that was operating under the legislative authority of the California Health and Safety Code § 2040. The field studies did not involve endangered or protected species. EVS traps were placed overnight and the collected mosquitoes were counted and identified to species using a dissection microscope. The timed mosquito collections over 24 h periods were made using the CBRT that was programed to rotate collection chambers every 3 h. Adult *Cx*. *tarsalis* were collected in a CBRT that was placed near Bair Island Ecological Reserve, CA USA (GPS coordinates: 37.501533, -122.216144). Mosquitoes that were collected for adult CDC bottle bioassays (BBA) or enzyme activity assays were transported in a humidified chamber and transferred to a nylon mesh chamber (24 x 14 x 13 cm) prior to use. A strain of *Cx*. *pipiens* mosquitoes that is susceptible to insecticides (strain SM-S1; *Cx*. *pipiens*^SM-S1^) was reared in an insectary using standard methods.

Meteorological data were obtained from a weather station that was located 1.3 km east and 3.2 km north of the Hayward Marsh using the US National Centers for Environmental Information database (www.ncdc.noaa.gov/cdo-web; [19]). Cumulative degree-days (DD) for each week were calculated as described previously for *Culex* mosquitoes [20] by comparing the daily average temperature to a baseline of 10°C (See Eq 1). If the DD calculation resulted in a negative value, zero was used instead.

$$\begin{array}{l} Equation\;for\;calculating\;DD\\ Weekly\;DD=\sum \left(over\;week\;number\right)\left[\left(T_{max}+T_{min}\right)/2\right]–T_{base},\;where\;T_{base}=10{}^{\circ}C \end{array}$$ Eq 1

### Adult bottle bioassays

Timed knock-down BBA were conducted to compare the insecticide resistance of adult *Cx*. *erythrothorax* to *Cx*. *pipiens*^SM-S1^, as previously described [21]. Field-collected *Cx*. *erythrothorax* were used because of known difficulty in laboratory colonization [22], as was done previously [23]. Briefly, the inside of clear 250 ml graduated media bottles (DWK Life Sciences LLC, Millville, NJ) were evenly coated with 1 ml of technical grade insecticide (permethrin, resmethrin, etofenprox, or naled; Chem Service, West Chester, PA) that was diluted in acetone. PBO (Chem Service, West Chester, PA) was dissolved with technical grade permethrin. Control bottles contained only the diluent or diluent with PBO. The diluent was evaporated from the interior of the bottles at room temperature using a gentle steam of nitrogen gas. Adult female mosquitoes were transferred to the bottles (n > 25 mosquitoes per bottle), and the number of dead or knocked down mosquitoes were recorded every 15 min for 90 min (N = 3–7 replicate bottles for each insecticide concentration). A mosquito was recorded as dead or knocked down if it could not stand unaided when the bottle was gently rotated; otherwise, the mosquito was counted as alive. The percent mortality was reported (i.e. the proportion dead or knocked down at the 90 min time point). Resistance ratios were calculated using LC_50_ values with those from *Cx*. *pipiens*^SM-S1^ in the denominator.

### Enzyme activity assays

Individual adult *Cx*. *erythrothorax* or *Cx*. *pipiens*^SM-S1^ that had not been exposed to insecticide were placed a microcentrifuge tube that contained a 5 mm glass bead, homogenized in potassium phosphate buffer chilled to 4°C using a Bead Mill 24 (Fisher Scientific, Waltham, MA) for 25 s at a speed setting of 4.0, and the homogenate clarified using centrifugation (3 min, 10,000 x g). Enzyme assays were conducted as described previously to evaluate the activity of MFO [24], GST [25], alpha-esterase [26], beta-esterase [26], and for the insensitive acetylcholinesterase assay [27]. To normalize the enzyme activity data to account for differences in mosquito size, the protein content of each mosquito homogenate was determined using a Pierce BCA protein assay kit, as described by the manufacturer (Thermo Scientific, Waltham, MA). Absorbances were measured for MFO at 620 nm, GST at 340 nm, alpha- and beta-esterase at 540 nm, and insensitive acetylcholinesterase at 414 nm using an Epoch Microplate Spectrophotometer (BioTek Instruments, Winooski, VT). Enzyme activity was reported as the absorbance value divided by the micrograms of protein in each mosquito homogenate.

### Larvicide application and vegetation removal for mosquito control

Approximately 100 kg of Vectolex FG (Clarke, St. Charles IL), VectoMAX FG (Valent Bio Sciences, Libertyville, IL), or Vectobac G (Valent Bio Sciences, Libertyville, IL) larvicide was applied at the Hayward Marsh near the edges of emergent bulrush vegetation every 1–3 weeks at the maximum label rate of 22 kg / hectare using a Mist Duster MD 155DX powered backpack blower (Maruyama US, Fort Worth, TX) by walking the perimeter of the marsh or by boat to access the emergent vegetation that was not adjacent to the shoreline. The active ingredient(s) of the larvicides were: *Bacillus sphaericus* for Vectolex FG, *Bacillus sphaericus* and *Bacuillus thuringiensis subsp*. *israelensis* for VectoMAX FG, and *Bacuillus thuringiensis subsp*. *israelensis* for Vectobac G. One product was used during each application week and the products were rotated in the order indicated above. Mosquito abundance was evaluated every 1–3 weeks at Hayward Marsh using 3–10 EVS traps that were baited with dry ice. Emergent vegetation was removed from the site between weeks 40–50 of 2016 using an Aquamog SRX-109 (Aquatics Unlimited, Martinez, CA). The quantity of vegetation that was removed was evaluated using aerial imagery, courtesy of the U.S. Geological Survey, by drawing bounding boxes around the regions where the vegetation was removed using Adobe Photoshop CC (Version 21.1.2; Adobe, San Jose, CA).

### Statistical methods

Data was plotted and analyzed using Prism software (version 8.3.0; GraphPad Software, San Diego, CA). Each insecticide concentration was assessed in triplicate BBA for *Cx*. *erythrothorax* and *Cx*. *pipiens*^SM-S1^, and the mean ± the standard error of the mean (SEM) was calculated. The lethal concentration of pesticide that knocked down or killed 50% of the mosquitoes (LC_50_) in BBA was calculated from the equation of the line that was generated from a linear regression of the dose-mortality data for each pairing of species and pesticide. The time at which 50% of the mosquitoes were knocked down (KDT_50_) was calculated from the equation of the line from the timed BBA. The slope and intercepts of linear regressions were evaluated with an analysis of covariance. Enzyme activity assays were conducted in triplicate for each mosquito homogenate (n = 10 mosquitoes per species). Activities of each enzyme in the two mosquito species were compared with unpaired t test.

## Results and discussion

### Adult activity periods of *Cx*. *erythrothorax* relative to *Cx*. *tarsalis*

**Fig 1:**
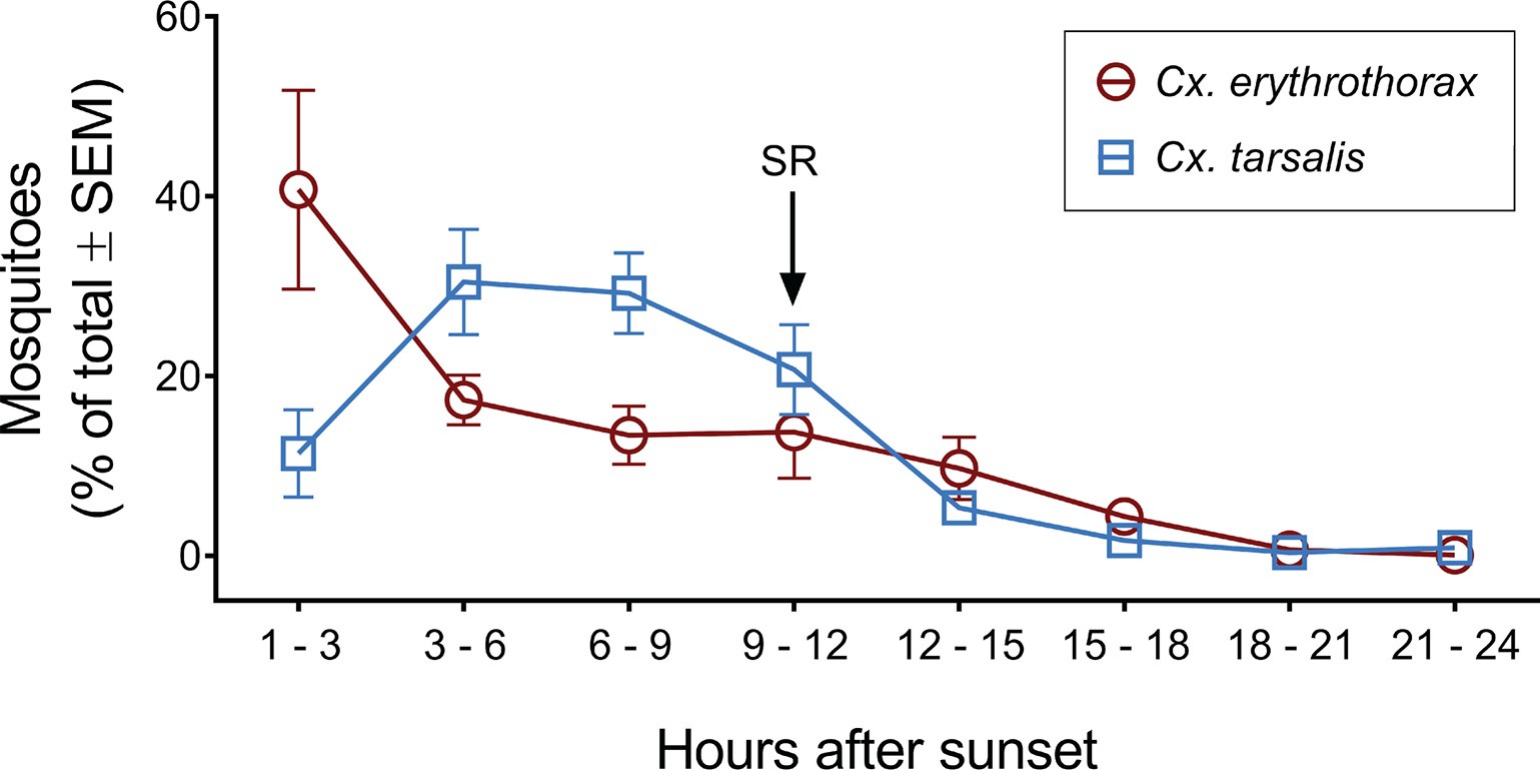
Hourly abundance of *Cx*. *erythrothorax* and *Cx*. *tarsalis* in a marsh habitat. SR indicates the collection time period that sunrise occurred.

The Collection Bottle Rotator Trap (CBRT) was operated over three consecutive 24 h periods and captured 10041 ± 5332 *Cx*. *erythrothorax* and 267 ± 74 *Cx*. *tarsalis* per day (Fig 1). The adult female mosquitoes that were collected in CBRT peaked at 1–3 h after sunset for *Cx*. *erythrothorax* while *Cx*. *tarsalis* were most abundant 3–6 h after sunset (Fig 1; 5527 ± 4070 *Cx*. *erythrothorax* and 83 ± 30 *Cx*. *tarsalis*). Adult mosquito abundance decreased markedly for both species during the 12–15 h collection period, which occurred immediately after sunrise (Fig 1). The nocturnal bloodmeal-seeking by female *Cx*. *erythrothorax* was similar to *Cx*. *tarsalis*, *Cx*. *pipiens* and *Anopheles gambiae* [7, 28, 29] and correlated with nighttime roosting of waterfowl in the marsh.

### Bioassays evaluating insecticide resistance

A CDC bottle bioassay was used to assess permethrin susceptibility of *Cx*. *erythrothorax* that were collected from a marsh habitat that supports diverse wildlife. Mosquito knock down or mortality in bottles that contained only diluent or PBO was less than 4% (not shown). *Culex erythrothorax* was 9-fold more sensitive to permethrin relative to *Cx*. *pipiens*^SM-S1^ (Table 1; resistance ratio (RR) of 0.11). A RR ≤ 1 indicates that the insects under evaluation are as susceptible to an insecticide as a strain with known high susceptibility (*Cx*. *pipiens*^SM-S1^ in the current study). There was no difference in the slopes of the linear regression lines for both species exposed to permethrin, suggesting that their biological responses to permethrin were similar (F (1,5) = 2.492, P = 0.1753). However, the y-intercepts of the lines were different, indicating that the field-caught *Cx*. *erythrothorax* were intrinsically more sensitive to permethrin relative to *Cx*. *pipiens*^SM-S1^ (y-intercept of 68.3 ± 13.3 and 101.0 ± 15.0, respectively; F (1,6) = 10.73, P = 0.0169). The *Cx*. *erythrothorax* also displayed high sensitivity to the pyrethroid etofenprox, and to the organophosphate insecticide naled (Table 1; RR of 0.50 and 0.18, respectively). The RR value for resmethrin was high (2.8), which taken alone would suggest that the mosquitoes were resistant to that insecticide [30, 31]. However, the LC_50_ value for resmethrin in both species was low and similar to the LC_50_ values of permethrin and naled for *Cx*. *erythrothorax* (Table 1). There was no difference in the mortality of *Cx*. *erythrothorax* and *Cx*. *pipiens*^SM-S1^ in BBAs with 1 μg of resmethrin, the highest concentration tested (unpaired t-test, P = 0.5845), suggest unusually high susceptibility of *Cx*. *pipiens*^SM-S1^ to resmethrin.

**Table 1:** Susceptibility of adult female *Cx*. *erythrothorax* and *Cx*. *pipiens*^SM-S1^ to insecticides using a CDC bottle bioassay.

Insecticides	Species	Range (μg) and number (N) of insecticide concentrations tested	Equation of linear regression	R squared	LC_50_
**Permethrin**	*Culex erythrothorax*	0.125–1.0 (5)	Y = 55.49*X + 30.52	0.6999	0.35
	*Culex pipiens*^SM-S1^	0.75–6.0 (4)	Y = 17.13*X—1.985	0.9142	3.0
**Resmethrin**	*Culex erythrothorax*	0.125–1.0 (4)	Y = 100.7*X—18.63	0.9718	0.68
	*Culex pipiens*^SM-S1^	0.125–1.0 (5)	Y = 63.42*X + 34.51	0.7805	0.24
**Etofenprox**	*Culex erythrothorax*	0.25–8.0 (5)	Y = 11.03*X + 5.255	0.9441	4.1
	*Culex pipiens*^SM-S1^	2.0–20.0 (4)	Y = 3.829*X + 18.92	0.6989	8.1
**Naled**	*Culex erythrothorax*	0.1–2.0 (5)	Y = 38.12*X + 22.76	0.8033	0.71
	*Culex pipiens*^SM-S1^	2.0–6.0 (3)	Y = 21.93*X—39.14	0.9997	4.1

Resistance to pyrethroids can occur quickly, sometimes within six generations [32]. The high susceptibility of the adult *Cx*. *erythrothorax* to insecticides that are commonly used for vector control suggests that the mosquitoes had not been exposed previously in the wetland habitat to sufficient levels of insecticide to induce resistance. Mosquito larvae that are exposed to pollutants for ten generations also display increased resistance to permethrin [33], presumably due to increased activity of enzymes that detoxify both the pollutant and insecticide. Surveying mosquitoes in marshland habitats for insecticide resistance may provide a sensitive and cost-effective means for determining if insecticide runoff or other pollutants are present in the habitat. The high susceptibility of *Cx*. *erythrothorax* suggest that the habitat from where they were collected contains very low levels of pollutants or insecticides.

Exposing adult *Cx*. *erythrothorax* for 90 min to 5 μg or 20 μg of PBO in the absence of permethrin did not affect mortality relative to treatments that lacked PBO (Table 2; unpaired t test, P = 0.5048 and 0.7452, respectively). Inclusion of 5 μg or 20 μg of PBO to bottles containing 0.5 μg of permethrin increased mosquito mortality by 2.0- and 2.3-fold (unpaired t tests, P = 0.0346 and 0.0257), respectively (Table 2). Neither of the PBO concentrations affected mortality of *Cx*. *erythrothorax* that were exposed to bottles containing 2.0 μg of permethrin (Table 2; unpaired t tests, P = 0.5478 and 0.1321, respectively). The mosquitoes were both highly susceptible to permethrin (Table 1) and PBO did not substantially increase the mortality of mosquitoes that were exposed to the highest concentration of permethrin that was evaluated (Table 2). When organic crops are near to where mosquito control is needed and a BBA shows the mosquitoes are susceptible to permethrin, botanical insecticides such as pyrethrins that are formulated without PBO should be effective against *Cx*. *erythrothorax*.

**Table 2:** Mortality of *Cx*. *erythrothorax* exposed to permethrin with or without the synergist PBO (N = 6 BBA per treatment).

Permethrin concentration (μg)	PBO concentration (μg)	Mortality (% ± SEM)
0	0	4.5 ± 1.3
	5	1.9 ± 2.3
	20	4.5 ± 1.8
0.5	0	21.8 ± 7.5
	5	42.9 ± 2.4
	20	50.9 ± 6.8
2.0	0	82.3 ± 4.9
	5	77.1 ± 5.9
	20	92.6 ± 2.9

**Fig 2:**
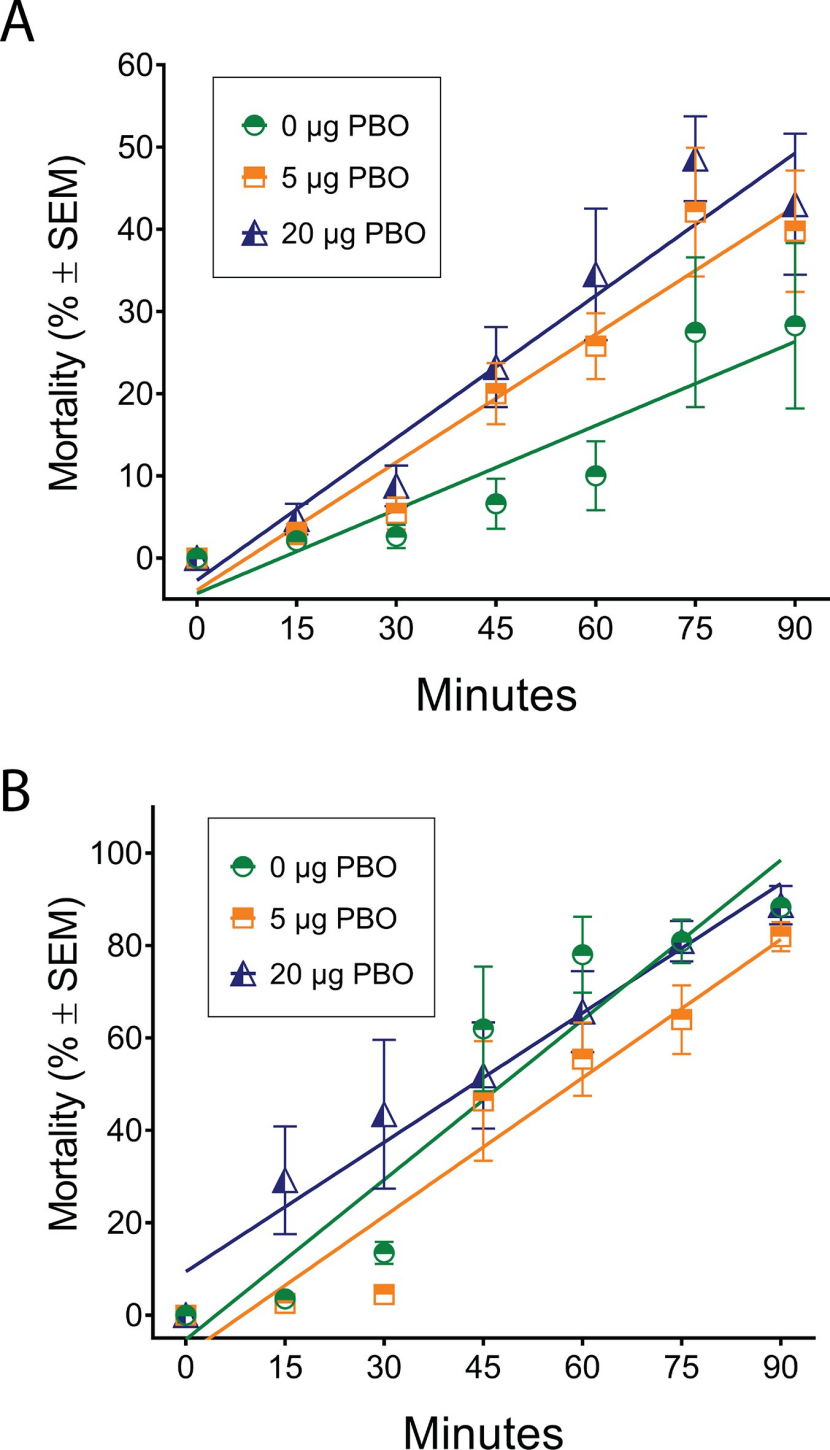
Mortality of *Cx*. *erythrothorax* after exposure to permethrin with or without PBO in a bottle bioassay. The average mortality of *Cx*. *erythrothorax* at 15 min intervals after exposure to 0.5 μg of permethrin (2A) or 2.0 μg of permethrin (2B) in the presence of 0, 5 or 20 μg of PBO (0.5 μg permethrin: 0 μg PBO, Y = 0.3403*X—4.302, R^2^ = 0.8483; 5 ug PBO: Y = 0.5186*X—3.907, R^2^ = 0.9356; 20 ug PBO: Y = 0.5774*X—2.711, R^2^ = 0.9327; 2.0 μg permethrin: 0 μg PBO, Y = 1.153*X– 5.291, R^2^ = 0.9041; 5 μg PBO, Y = 0.9989*X– 8.582, R^2^ = 0.9265; 20 μg PBO, Y = 0.9332*X + 9.413, R^2^ = 0.9678).

Mortality increased linearly from 15–90 min for mosquitoes exposed to 0.5 μg of permethrin and adding 5 μg of PBO did not affect the mortality rate (Fig 2A; F (1,80) = 3.861, P = 0.0529). The 20 μg PBO + 0.5 μg permethrin treatment increased the mortality rate relative to the 0.5 μg permethrin treatment (Fig 2A; F (1, 80) = 6.054, P = 0.0160). The KDT_50_ was reduced by 43% when 5 μg of PBO was added to the 0.5 μg permethrin treatment and by 54% when 20 μg of PBO was included compared to the 0.5 μg permethrin treatment that lacked PBO (Fig 2A; KDT_50_ for 0.5 μg permethrin treatments: 160 min for 0 μg PBO, 104 min for 5 μg PBO, and 91 min for 20 μg PBO). PBO did not affect the rate of mortality for mosquitoes exposed to 2.0 μg of permethrin (F (2,120) = 1.252, P = 0.2898). The KDT_50_ for the 2.0 μg permethrin treatments were lower than those for the 0.5 μg permethrin treatments (Fig 2A and 2B). In aggregate, the results demonstrate that *Cx*. *erythrothorax* were exquisitely sensitive to permethrin, and that inclusion of 5 or 20 μg of PBO with 0.5 μg permethrin increased the sensitivity of the mosquitoes to permethrin and hastened knockdown.

As people build increasingly closer to wetlands, it is likely that highly abundant mosquitoes such as *Cx*. *erythrothorax* will migrate into these communities to acquire a bloodmeal before returning to the wetland to oviposit. Many bird species that utilize wetland habitats are reservoirs for WNV [34]. Adult *Cx*. *erythrothorax* likely become infected with arboviruses while they are in marsh habitats when viremic birds are present. The sympatric distribution of abundant *Cx*. *erythrothorax* and marsh birds that are susceptible to WNV with people that live, work and recreate near wetlands may increase the potential for WNV transmission to people. Pyrethroids are toxic to many aquatic organisms, including fish and should not be applied to areas where surface water is present, such as marsh habitats [35]. However, permethrin in conjunction with the synergist PBO could be effectively employed by public health agencies to control viremic *Cx*. *erythrothorax* that enter human communities when seeking bloodmeals.

### Activity of insecticide metabolizing enzymes

**Fig 3:**
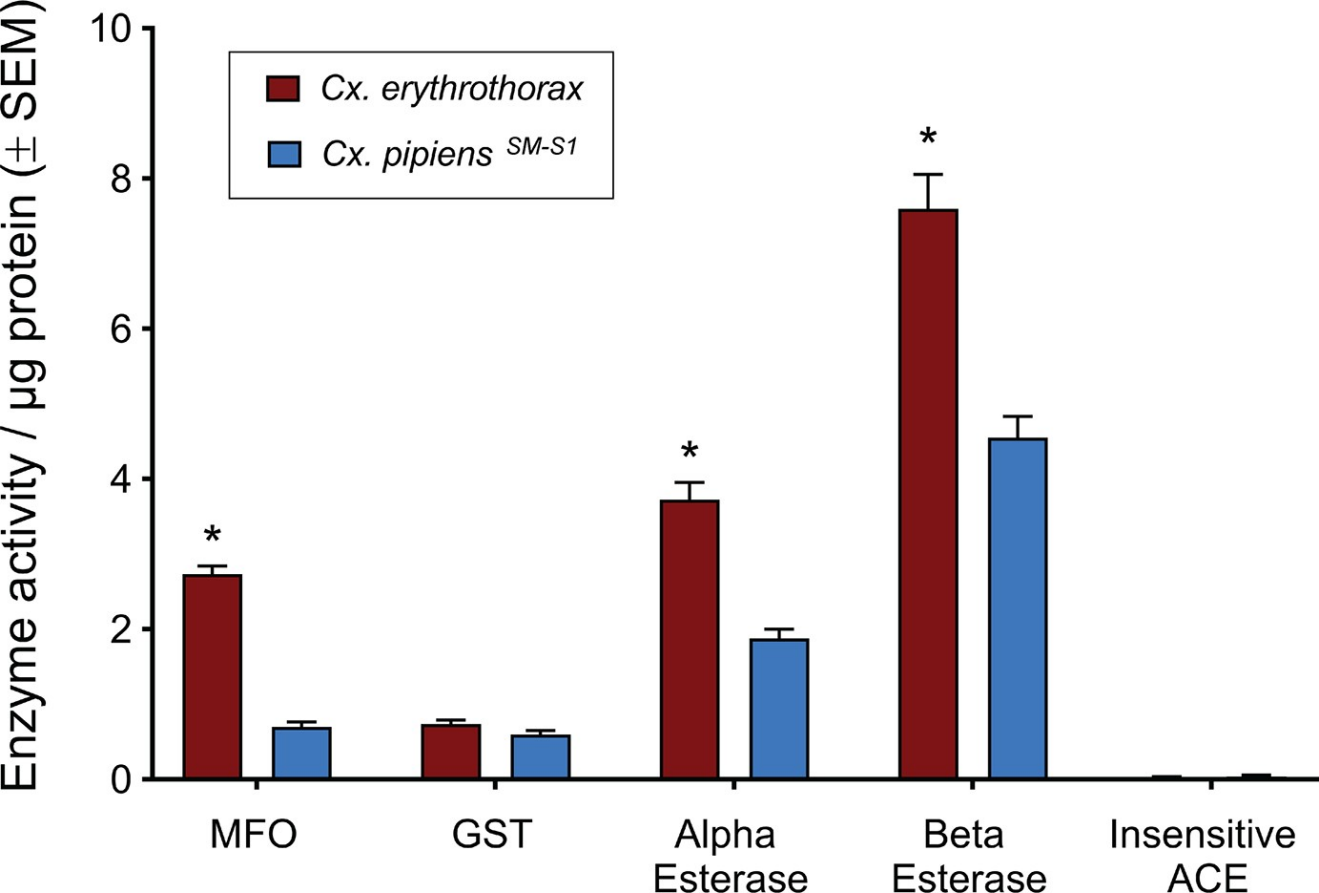
Activity of insecticide detoxifying enzymes normalized to the quantity of mosquito protein. Asterisks (*) indicate significant differences in the activity of an enzyme between the species.

The activity of detoxifying enzymes in homogenates of individual mosquitoes from *Cx*. *erythrothorax* were compared to those from the susceptible reference strain to determine if insecticides could be potentially metabolized and inactivated. There was no difference in the activity of GST between *Cx*. *erythrothorax* and *Cx*. *pipiens*^SM-S1^, and insensitive acetylcholinesterase was not detected in either species (Fig 3, unpaired t test, P = 0.0932). The *Cx*. *erythrothorax* displayed higher enzyme activity for MFO, alpha-esterase, and beta-esterase relative to *Cx*. *pipiens*^SM-S1^ (Fig 3; unpaired t tests, P < 0.05). However, *Cx*. *erythrothorax* were more sensitive than *Cx*. *pipiens*^SM-S1^ to several insecticides (Table 1). The higher enzyme activity in *Cx*. *erythrothorax* may be present to metabolize plant phytochemicals or environmental toxins that leach into the marsh which would be absent in the water used to grow *Cx*. *pipiens*^SM-S1^ in a laboratory environment [36]. The KDT_50_ values for *Cx*. *erythrothorax* exposed to permethrin in BBA (Fig 2) were higher than what has been reported previously for *Culex spp*. that were resistant to permethrin [37–39]. This delayed mortality (Fig 2) may have resulted from the elevated activity of detoxifying enzymes (Fig 3) or unmeasured factors such as differences in cuticular structure or chemistry that contributed to elevating the KDT_50_ values [40]. An increased capacity to detoxify may permit mosquitoes that are exposed to insecticides in the field sufficient time to escape the application site and survive.

### Mosquito control: Larvicide applications and vegetation removal

**Fig 4:**
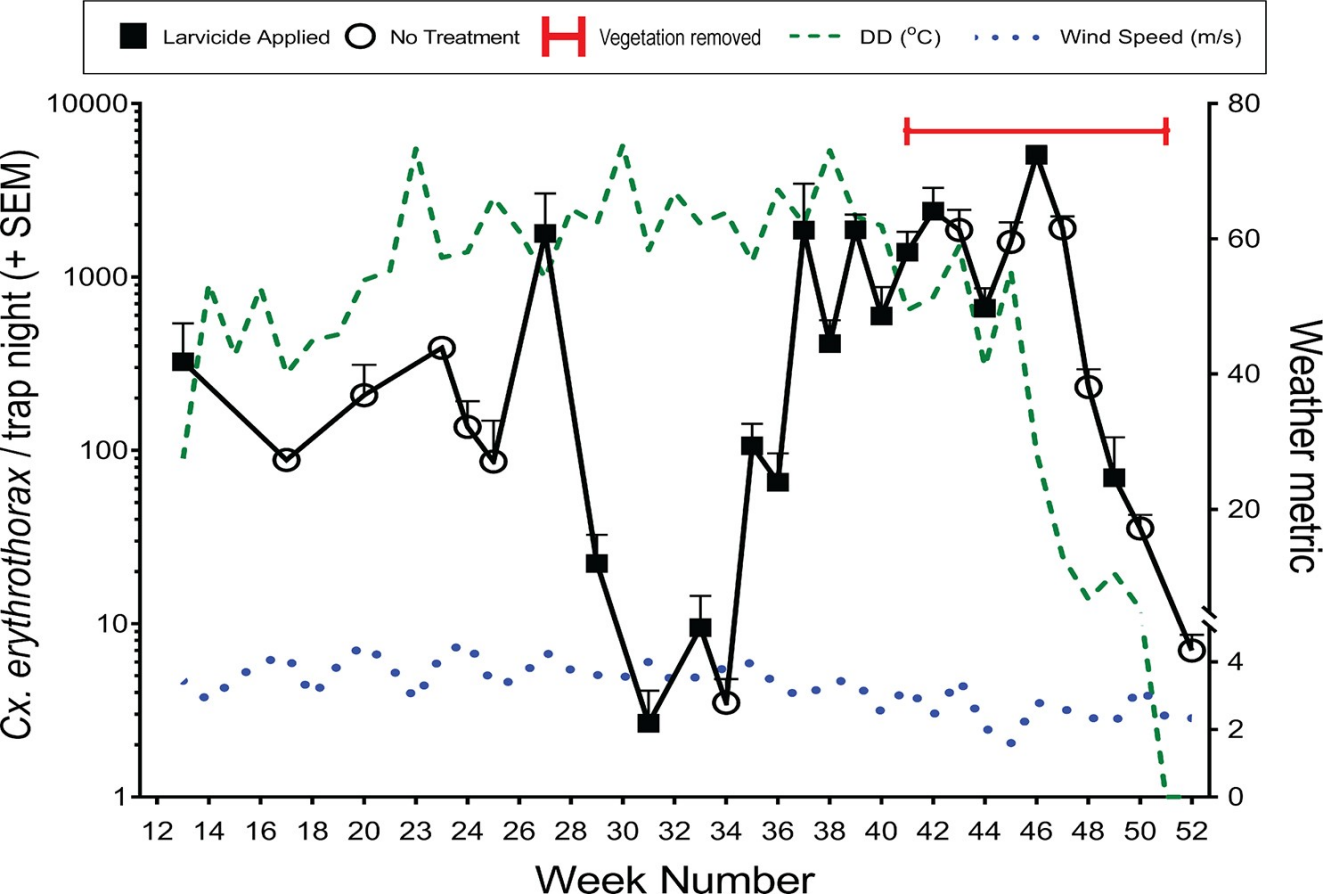
Average weekly abundance of *Cx*. *erythrothorax* in a marsh habitat relative to wind speed and Degree Days (DD).

From week 13–52 of 2016, the abundance of adult female *Cx*. *erythrothorax* ranged from 2.7–5034 mosquitoes per trap night and averaged 827 ± 156 mosquitoes per trap night (Fig 4). Average weekly wind speeds did not differ substantially over the study period (Fig 4), suggesting that mosquito abundance was not affected by the ability of EVS traps to capture mosquitoes or displacement of the mosquitoes from the marsh by wind.

Because abundance exceeded 1000 mosquitoes / trap night during week 27, the impact of applying larvicide and removing emergent vegetation on mosquito abundance was evaluated. Although the adult *Cx*. *erythrothorax* from the study site were highly susceptible to adulticides (Table 1), the labels on those insecticides prohibit their application over marshes. Therefore, we were unable to assess the impact of adult-targeting insecticides on mosquito abundance at the study site. To determine if larvicides could reduce mosquito abundance, approximately 22 kg/ha of larvicide was applied at the site every 1–2 weeks from week 27–46 (Fig 4). Changes in weekly DD tracked with mosquito abundance, with the exception of the period between week 29–34 when mosquito abundance was low while DD was high (Fig 4), suggesting that the larvicide during this period may have contributed to reducing mosquito abundance.

**Fig 5:**
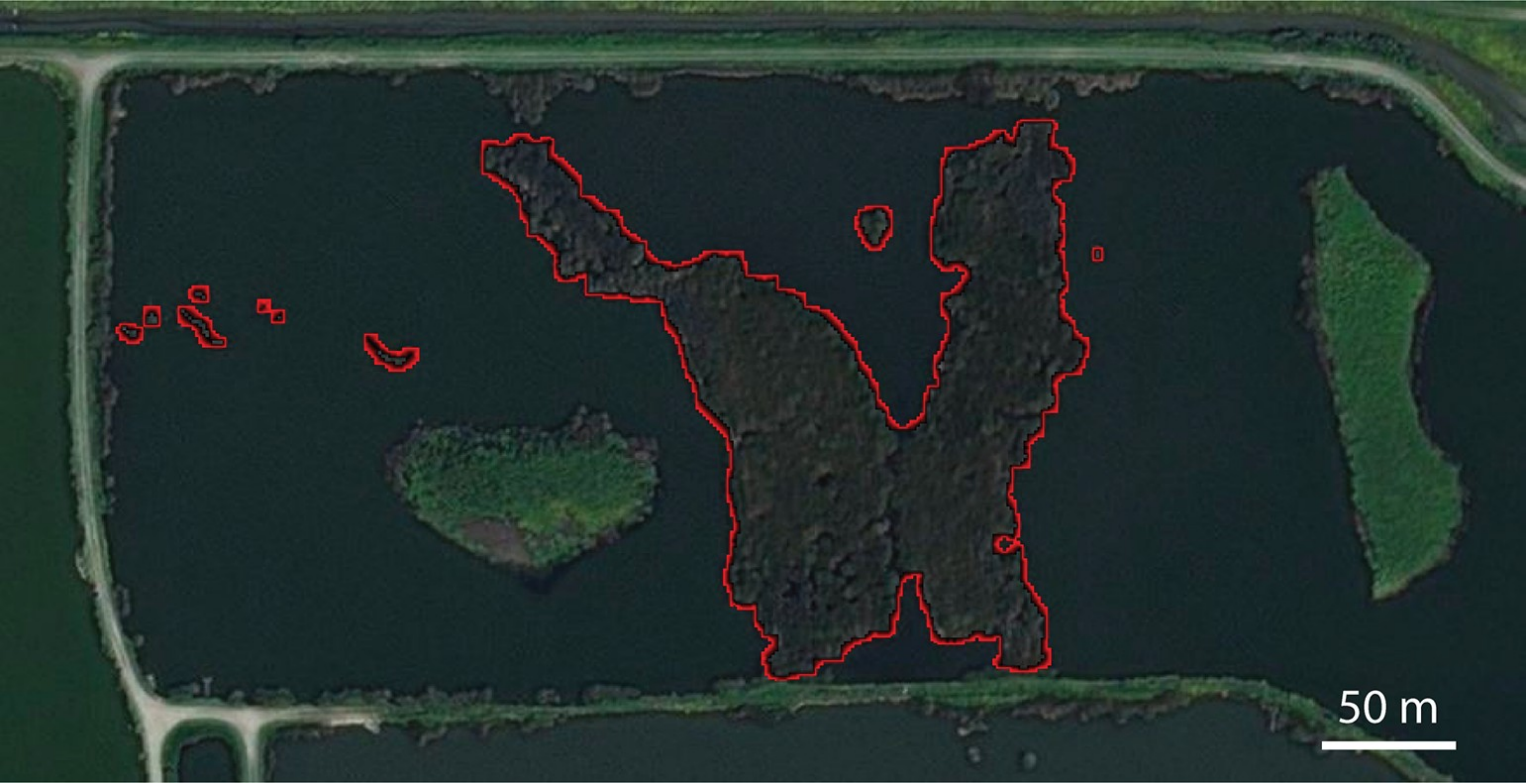
Aerial imagery of the site before removing emergent vegetation. The emergent vegetation that was removed is highlighted in red. Map data: U.S Geological Survey, Department of the Interior/USGS.

Although larvicide continued to be applied at the site during weeks 37–42, mosquito abundance remained high (Fig 4; 1421 ± 290 mosquitoes / trap night). Bands of emergent vegetation that are 20 m wide support high *Cx*. *erythrothorax* abundance even when larvicide is applied [41]. Because the width of emergent vegetation at the Hayward Marsh exceeded 80 m near the center of the marsh (Fig 5), the larvicide may not have penetrated the vegetation and entered the water to impact the mosquito larvae that were *in situ*. To reduce the suitability of the habitat for *Cx*. *erythrothorax*, approximately 1.5 x 10^4^ m^2^ of emergent vegetation was removed from the marsh during weeks 41–51 of 2016, reducing the maximum width of the emergent vegetation to 7 m at the periphery of the march (Fig 5). The abundance of adult *Cx*. *erythrothorax* was reduced substantially as the emergent vegetation was reduced, however it was coincident with concomitant larvicide applications and a reduction in DD (Fig 4).

The abundance of *Cx*. *erythrothorax* is typically underrepresented in larval surveys of wetland habitats [42]. The efficacy of mosquito control measures was previously assessed by measuring adult abundance of this species using CO_2_-baited EVS traps [9]. Therefore, we used CO_2_-baited EVS traps to assesses the impact of larvicide applications and vegetation removal in a wetland marsh habitat on adult *Cx*. *erythrothorax* abundance. The nearest marsh habitat with emergent vegetation that could support *Cx*. *erythrothorax* and had a surface area greater than 0.25 hectares was 6.7 km distal from the study site (GPS coordinates: 37.582500, -122.088729). Dispersion distances of adult *Cx*. *erythrothorax*, measured using mark-recapture, are typically less than 0.5 km and no greater than 2 km from the release site [6]. Therefore, the mosquitoes that were collected in the traps for the present study likely emerged from study site marshland.

The study to evaluate the impact of larvicide application and vegetation removal lacked a control site where interventions were not made because people reside near or utilize each of marsh habitats in the area. Allowing uncontrolled growth of mosquitoes that can transmit WNV to people in such areas would be unethical. As a proxy for a control site, we measured adult mosquito abundance at the study site using CO_2_-baited EVS traps for three years after the interventions (i.e. during 2017–2019). Adult *Cx*. *erythrothorax* abundance was on average 16-fold lower during the three years after the intensive larvicide applications and vegetation removal (89.4 ± 247 mosquitoes / trap night; N = 83 trap nights; t test, P < 0.001), suggesting that the interventions had multi-year benefits.

When faced with unacceptably high abundance of *Cx*. *erythrothorax* in a marsh, land managers may consider forgoing intensive and costly larvicide applications if the width of emergent vegetation is high, and instead focus on removing the vegetation. Although costly, by doing so, subsequent larvicide applications are more likely to reduce the growth of mosquito larvae while increasing the ability of fish and invertebrates to prey upon the mosquito larvae. The biomass of a single *Schoenoplectus* plant (*i*.*e*. bulrush) can increase by 0.5–3.3 kg in a single year [43, 44], pointing to the importance of implementing an ongoing vegetation management program in mash habitats that abut urban and suburban areas to keep *Cx*. *erythrothorax* abundance low.

## Conclusions

In conclusion, the adult *Cx*. *erythrothorax* were highly susceptible to several insecticides even though the activity of detoxifying enzymes was elevated. The time of day that *Cx*. *erythrothorax* were most active coincides with that of *Cx*. *tarsalis*, *Cx*. *pipiens* and *Cx*. *quinquefasciatus*, each of which can transmit arboviruses such as WNV to people. Thus, efforts to control viremic *Cx*. *erythrothorax* in areas that surround marsh habitats may also be effective against these and other crepuscular *Culex* species. Although larvicide applications likely reduced adult *Cx*. *erythrothorax* populations, the impact was short lived, and the effort had a high financial cost. An effective approach for controlling *Cx*. *erythrothorax* larvae in a marsh with dense emergent vegetation is to remove the dense vegetation [45]. By doing so, the habitat can no longer provide the environmental conditions needed by *Cx*. *erythrothorax*, and would allow larvicide that is applied to enter the water column where the mosquitoes live.
